# Mesenchymal stromal cell secretory molecules improve the functional survival of human islets

**DOI:** 10.1111/dme.15227

**Published:** 2023-10-03

**Authors:** Tzu‐Wen Hong, Sara Caxaria, Lydia F. Daniels Gatward, Sufyan Hussain, Min Zhao, Aileen J. F. King, Chloe L. Rackham, Peter M. Jones

**Affiliations:** ^1^ Department of Diabetes, School of Cardiovascular and Metabolic Medicine and Sciences King's College London London UK; ^2^ William Harvey Research Institute, Barts and the London School of Medicine Queen Mary University of London London UK; ^3^ Department of Diabetes and Endocrinology, Guy's and St Thomas' NHS Foundation Trust London UK; ^4^ Exeter Centre for Excellence in Diabetes, Institute of Biomedical and Clinical Science University of Exeter Exeter UK

**Keywords:** apoptosis, GPCR, human islet, insulin secretion, islet transplantation, MSCs, type 1 diabetes

## Abstract

**Aims:**

Human islet transplantation as a therapy for type 1 diabetes is compromised by the loss of functional beta cells in the immediate post‐transplantation period. Mesenchymal stromal cells (MSCs) and MSC‐derived secretory peptides improve the outcomes of islet transplantation in rodent models of diabetes. Here, we utilized a mouse model for human islet transplantation and assessed the effects of a cocktail of MSC‐secreted peptides (screened by MSC‐secretome for human islet GPCRs) on the functional survival of human islets.

**Methods:**

Human islets from nine donors (Age: 36–57; BMI: 20–35) were treated with a cocktail of human recombinant annexin A1 (ANXA1), stromal cell‐derived factor‐1 (SDF‐1/CXCL12) and complement component C3 (C3a). Glucose‐stimulated insulin secretion (GSIS) was assessed in static incubation, and cytokine‐induced apoptosis was assessed by measuring caspase 3/7 activity. mRNA expression levels were determined by qPCR. Human islet function in vivo was assessed using a novel model for human islet transplantation into a T1D mouse model. Human islet function in vivo was assessed using islet transplantation under the kidney capsule of immunodeficient mice prior to STZ destruction of endogenous mouse beta cells to model T1DM.

**Results:**

Pretreatment with a cocktail of MSC‐secreted peptides increased GSIS in vitro and protected against cytokine‐induced apoptosis in human islets isolated from nine donors. Animals transplanted with either treated or untreated human islets remained normoglycaemic for up to 28 days after STZ‐administration to ablate the endogenous mouse beta cells, whereas non‐transplanted animals showed significantly increased blood glucose immediately after STZ administration. Removal of the human islet graft by nephrectomy resulted in rapid increases in blood glucose to similar levels as the non‐transplanted controls. Pretreating human islets with the MSC‐derived cocktail significantly improved glucose tolerance in graft recipients, consistent with enhanced functional survival of the treated islets in vivo.

**Conclusion:**

Pretreating human islets before transplantation with a defined cocktail of MSC‐derived molecules could be employed to improve the quality of human islets for transplantation therapy for type 1 diabetes.

**What's new?**

- In this study, we aimed to determine whether our previous studies on the beneficial effects on mouse islets of mesenchymal stromal cell (MSC)‐derived secretory products could translate to clinically relevant human islets.
- Our results demonstrate that a cocktail of MSC‐derived secretory products improved human islet function in vitro, by increasing glucose‐stimulated insulin secretion and protecting against inflammatory cytokine‐induced apoptosis. Pretreatment of human islets with the MSC‐derived cocktail also improved their ability to maintain normoglycaemia in vivo.
- Our study suggests that pretreating isolated human islets with a defined cocktail of recombinant G‐protein coupled receptors ligands prior to transplantation will improve their functional survival and thus the clinical outcomes of human islet transplantation as a therapy for T1D.

## 1 INTRODUCTION

Allogeneic human islet transplantation is currently used to treat selected people with type 1 diabetes (T1D), but its widespread adoption is restricted by the limited availability of donor islets; by the loss of isolated islets during the isolation process; and by further extensive loss of islet function during the immediate post‐transplantation period (24–48 h) when islet survival is compromised by the hypoxic, inflammatory host environment.^1^, ^2^ Interventions which improve the functional survival of islets before and after transplantation will improve the outcome of individual grafts and will also enable the limited pool of donor islets to treat more people with T1D.^2^, ^3^, ^4^, ^5^, ^6^, ^7^

Mesenchymal stromal cells (MSCs) are adult progenitor cells which play a major role in tissue repair by migrating to the site of injury and instigating repair processes which includes secreting biologically active molecules with anti‐inflammatory, immunomodulatory and angiogenic properties.^8^, ^9^, ^10^, ^11^ Co‐localisation of MSCs with islets improves islet functional survival in vitro and transplantation outcomes in animal models of T1D in vivo.^12^, ^13^, ^14^ We have previously demonstrated that some of these benefits can be obtained by simply treating isolated mouse islets with MSC‐derived secretory products before transplantation,^15^, ^16^, ^17^ suggesting a simple, non‐invasive and cell‐free way to enhance islet graft function while avoiding the requirement to co‐transplant MSCs, and thus any clinical risks associated with MSC transplantation.^18^, ^19^ We have used our analyses of G‐protein coupled receptors (GPCRs)^20^ in mouse and human islets and of the mouse and human MSC‐secretome to identify MSC‐derived GPCR ligands likely to influence beta cell function.^21^, ^22^ Our experimental studies in mouse models have characterized a “cocktail” of GPCR ligands – annexin A1 (ANXA1), stromal cell‐derived factor‐1 (SDF1 or CXCL12) and complement component C3 (C3a) – which improve mouse islet functional survival in an inflammatory environment in vitro,^23^ and improve the ability of pretreated islet grafts to maintain normoglycaemia in a mouse model of T1D.^23^

The current study aimed to assess the translational potential of these experimental observations by assessing the effects on human islets of pretreatment with the MSC‐derived cocktail of recombinant molecules to influence their functional survival in vitro; and to assess their subsequent ability to maintain normoglycaemia in a novel model of human islet transplantation into a T1D mouse host.

## 2 METHODS

### 2.1 Human islet isolation and culture

Human islets isolated from ethically approved heart‐beating donors (seven men/two women; Age: 36–57; BMI: 20–35) were supplied by King's College London Hospital Human Islet Unit according to previously described protocols.^24^ Islets (50%–90% purity; 50%–90% viability) isolated from nine donors were received within 48 h of pancreas harvest from cadaveric donors (donor information in Table 1). Human islets were cultured in human islet culture medium: CMRL Medium (11,530,037, Thermo Fisher) supplemented with 10% foetal bovine serum (FBS) and 100 U/mL penicillin plus 0.1 mg/mL streptomycin at 37°C/ 5% CO_2_.

**TABLE 1:** Information of the human islet donor.

No.	Date (dd/mm/yyyy)	Gender	Blood	Age	BMI	Purity (%)	Viability (%)
1	10/12/2019	Male	B+	47	21	70	90
2	15/01/2020	Female	A+	48	35	85	85
3	03/10/2020	Female	A+	52	29	50	50
4	05/10/2020	Male	O+	39	28	65	80
5	29/10/2022	Male	A+	48	30.32	75	85
6	13/11/2022	Male	O+ ve	43	27.76	60	80
7	25/11/2022	Male	O+	49	27	90	80
8	08/12/2022	Male	B+	57	26.9	70	80
9	14/02/2023	Male	O+	36	23.39	60	85

### 2.2 MSC‐secreted GPCR ligands

A cocktail of human recombinant annexin A1 (ANXA1, 5 nM, 3770‐AN, R&D Systems), stromal cell‐derived factor‐1 (SDF‐1, 10 nM, 350‐NS, R&D Systems) and complement component C3a (C3a, 10 nM, 3677‐C3, R&D Systems) was freshly prepared in human islet culture medium before each incubation.

### 2.3 Human islet function in vitro

#### 2.3.1 Cytokine‐induced apoptosis

Human islet apoptosis in response to inflammatory cytokines was assessed by measuring caspase 3/7 activity using a Caspase Glo assay (Promega, UK), as previously described.^23^ Briefly, human islets were pre‐cultured alone or with a cocktail of MSC‐derived recombinant factors for 72 h (as described in Section 2.2). For the final 20 h of the culture period, half of the islets were exposed to mixed cytokines (50 U/mL IL‐1beta, 1000 U/mL interferon‐gamma and 1000 U/mL tumour necrosis factor‐alpha (Peprotech, UK), while untreated islets served as controls. All islets were incubated in media supplemented with 2% FBS for this final 20 h period. Size‐matched islets were hand‐picked into groups of five per well and Caspase‐Glo 3/7 reagent was added. After 1 h, light emission was detected using a Turner Biosystems Veritas microplate luminometer (Promega, UK).

#### 2.3.2 Islet secretory function

Glucose‐stimulated insulin secretion (GSIS) was assessed by using static incubation as previously described.^23^ Briefly, isolated islets were pre‐incubated for 2 h in human islet culture medium supplemented with 2 mM glucose. Groups of five size‐matched human islets were transferred into 1.5 mL tubes and incubated (37°C, 1 h) in a bicarbonate‐buffered physiological salt solution supplemented with 2 mmol/L CaCl_2_ and 0.5 mg/mL BSA and either 2 (basal) or 20 mM (stimulatory) glucose for 1 h. Supernatant samples were taken and stored at −20°C until assayed for insulin content using an in‐house radioimmunoassay (RIA) as previously described.^25^ The islet pellets were lysed in acidified ethanol, sonicated, and then stored at −20°C for the assessment of the islet insulin content by RIA.

#### 2.3.3 Islet gene expression

RNA was extracted from human islets using TRIzol, as previously described.^26^ The purity and concentration of isolated RNAs were measured using a Nanodrop 1000 spectrophotometer. Quality approved RNA samples were stored at −80°C prior to reverse transcription of mRNAs.^26^ Qiagen QuantiTect primers were used to quantify the expression of individual mRNAs relative to the housekeeping genes (Beta‐actin, ACTB) using the ΔΔCt method.

### 2.4 Human islet function in vivo

Experiments were performed in accordance with UK legislation under the Animals (Scientific Procedures) Act 1986 Amendment Regulations. We utilised a human islet transplantation model modified from a previously published study.^27^ In this model, donor human islets are transplanted under the kidney capsule of immunodeficient mice and allowed to engraft and revascularize before the selective destruction of the endogenous mouse beta cells by administration of multiple low‐dose STZ, at which point the human islet graft assumes control of the mouse host glycaemia. The timeline of the in vivo transplantation study is illustrated in Figure 3a. Briefly, nine immunodeficient mice (NOD.Cg‐Prkdc^scid^ Il2rg^tm1Wjl/^SzJ, 8‐week‐old men, NOD‐*scid*, nu/nu) were allowed to acclimate for 2 weeks before surgery, and randomly distributed into different groups (non‐transplanted, transplanted control and transplanted cocktail). Overall, 800 human islets (pretreated with cocktail for 72 h or control) were transplanted to the right kidney capsule and allowed to engraft, revascularize and become functionally stable for 24 days before ablation of the endogenous mouse β‐cells by 5 consecutive low‐dose daily IP‐injections of the β‐cell toxin streptozotocin (STZ: 70 kg/mg body weight). STZ enters mouse β‐cells via the GLUT2 transporter, which is expressed at much lower levels, if at all, in human β‐cells, thus leaving the STZ‐resistant human β‐cells to maintain glycaemic control.

Body weight and non‐fasted blood glucose concentrations were measured every other day in the morning using an Accu‐Chek glucose meter (Roche) after a blood droplet was generated via a needle‐prick to the end of the tail. Plasma concentrations of human insulin were assessed using a human insulin ELISA (10‐1132‐01, Mercodia). Human islet graft function was assessed by glucose tolerance tests (GTT) 14 days after islet transplantation (pre‐STZ) and 14 days after the STZ‐injection (post‐STZ, 3 days before graft removal by nephrectomy). Mice were fasted for 6 h and their basal blood glucose concentrations were measured. Glucose was injected intraperitoneally (3 g/kg body weight), and blood glucose concentrations were measured at 15‐, 30‐, 60‐, 90‐ and 120‐min post‐injection.

### 2.5 Statistical analysis

Statistical analysis used Student's *t*‐test or analysis of variance (ANOVA), as appropriate. Two‐way repeated‐measurement ANOVA was used with Bonferroni's post hoc test to analyse repeated measurements in the same animal at different time points. A *p* < 0.05 was considered statistically significant. All data are expressed as means ± SEM. Sample size (*n* = 4–9) from different islet donor was used for statistical tests representing biological replicates, independent measurements of the population from separate experiments. Data obtained from different individuals were plotted using the Superplot method in Graphpad prism 9 as described previously.^28^

## 3 RESULTS

### 3.1 MSC‐derived cocktail pretreatment improved human islet function in vitro

To assess the function of the recombinant, MSC‐derived GPCR agonist cocktail on human islet functional survival, we measured GSIS and islet cell apoptosis in response to inflammatory cytokines in human islets isolated from nine separate donors (Table 1).

**FIGURE 1:**
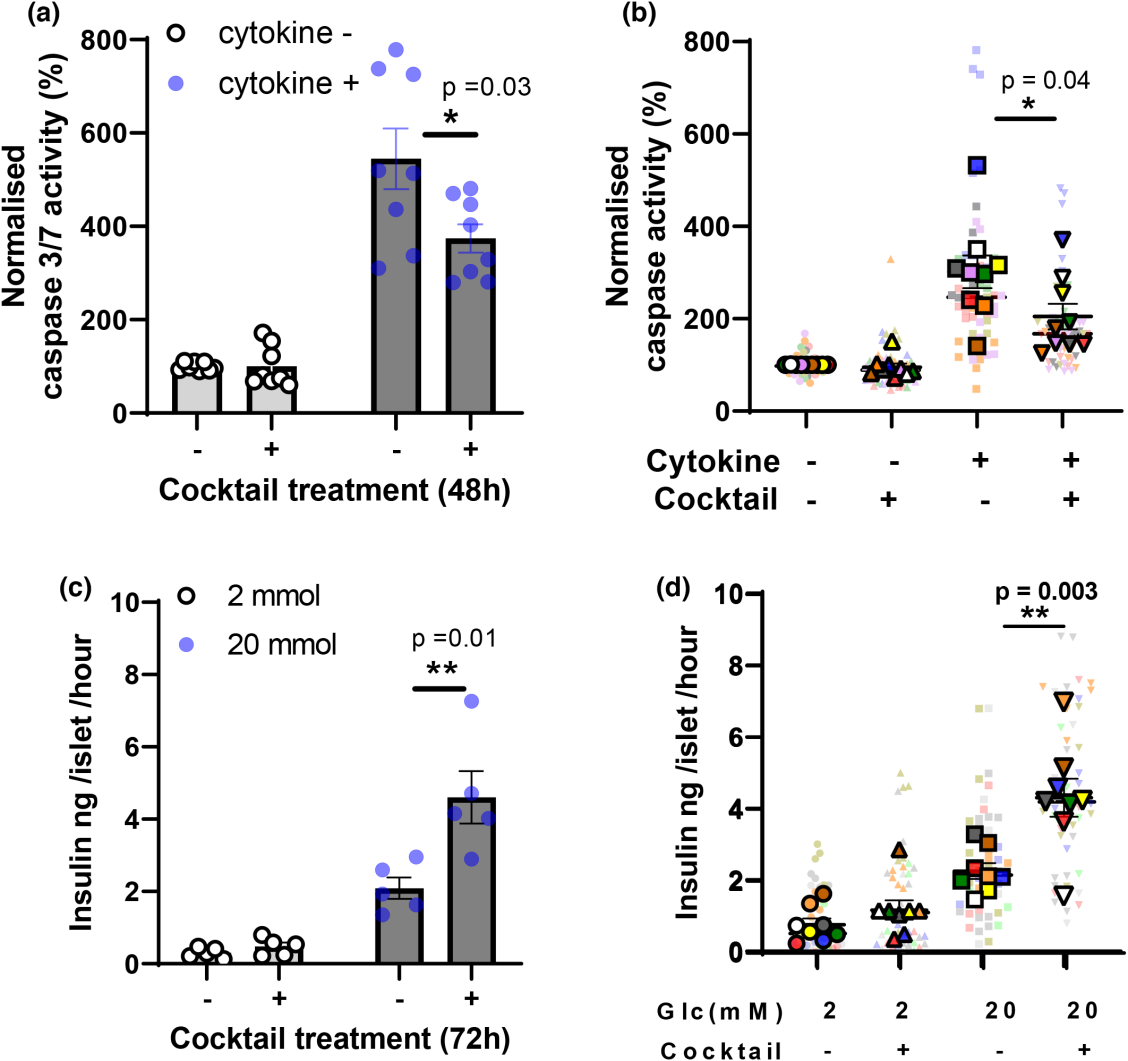
Mesenchymal stromal cell (MSC)‐derived cocktail pretreatment improved human islet function in vitro. (a, b) Pretreatment with the MSC‐derived cocktail protected human islet cells from inflammatory cytokine‐induced apoptosis. (a) Individual data from donor #5 (*n* = 8); (b) Superplot data from separate experiments using islets from nine individual donors. Data were analysed by Student's unpaired *t*‐tests: *p < 0.05, or **p < 0.01 cocktail pretreated versus untreated control islets. (c, d) Pretreatment with the MSC‐derived cocktail enhanced glucose‐induced insulin secretion from human islets. Insulin secretion was measured in static incubation (five islets per observation) in the presence of either 2 or 20 mM glucose, as shown. (c) Individual data from donor #5 (*n* = 5); (d) superplot data from separate experiments using islets from nine individual donors. Data were analysed by Student's unpaired *t*‐tests: **p < 0.01 cocktail pretreated versus untreated control islets. Same colour of two data points represents results from the same human donor as shown in Table 1.

Pretreatment with an MSC cocktail for 48 h protected the human islets from inflammatory cytokine‐ induced apoptosis, as shown in Figure 1a,b. Cocktail treatment had no effect on basal levels of apoptosis in the absence of cytokines, but significantly reduced cytokine‐induced apoptosis, as shown in the data using a single human islet preparation (donor 5; Table 1) in Figure 1a. Similar effects were observed using human islets isolated from seven of nine donors, with an overall statistically significant protective effect (*p* < 0.04) when data from all nine donors were pooled, as shown in Figure 1b.

Pretreatment with the MSC‐derived cocktail enhanced glucose‐stimulated (20 mM) insulin secretion in vitro, as shown in Figure 1c,d. Figure 1c shows data from a single islet donor (donor 5; Table 1) where cocktail treatment had no effect on basal (2 mM)‐induced insulin secretion, but significantly increased glucose stimulated (20 mM)‐insulin secretion. Similar effects were observed using human islets isolated from eight of nine donors, with an overall statistically significant protective effect (*p* < 0.003) when data from all nine donors were pooled (Figure 1d). Cocktail pretreatment caused a small increase in basal (2 mM glucose) insulin secretion in islets from six of eight donors but this did not achieve statistical significance when data from all nine donors were pooled (*p* = 0.2). The Super Plots in Figures 1b,d show individual (mean) data from the nine human islet donors represented in the same colours as shown in Table 1 for identification.

Together, these effects on isolated human islets of pretreatment with a defined cocktail of recombinant GPCR agonists secreted by human MSCs are consistent with the beneficial effects we have previously reported using mouse islets.^23^

### 3.2 MSC‐derived cocktail pretreatment increased insulin mRNA and protein content of human islets function in vitro

**FIGURE 2:**
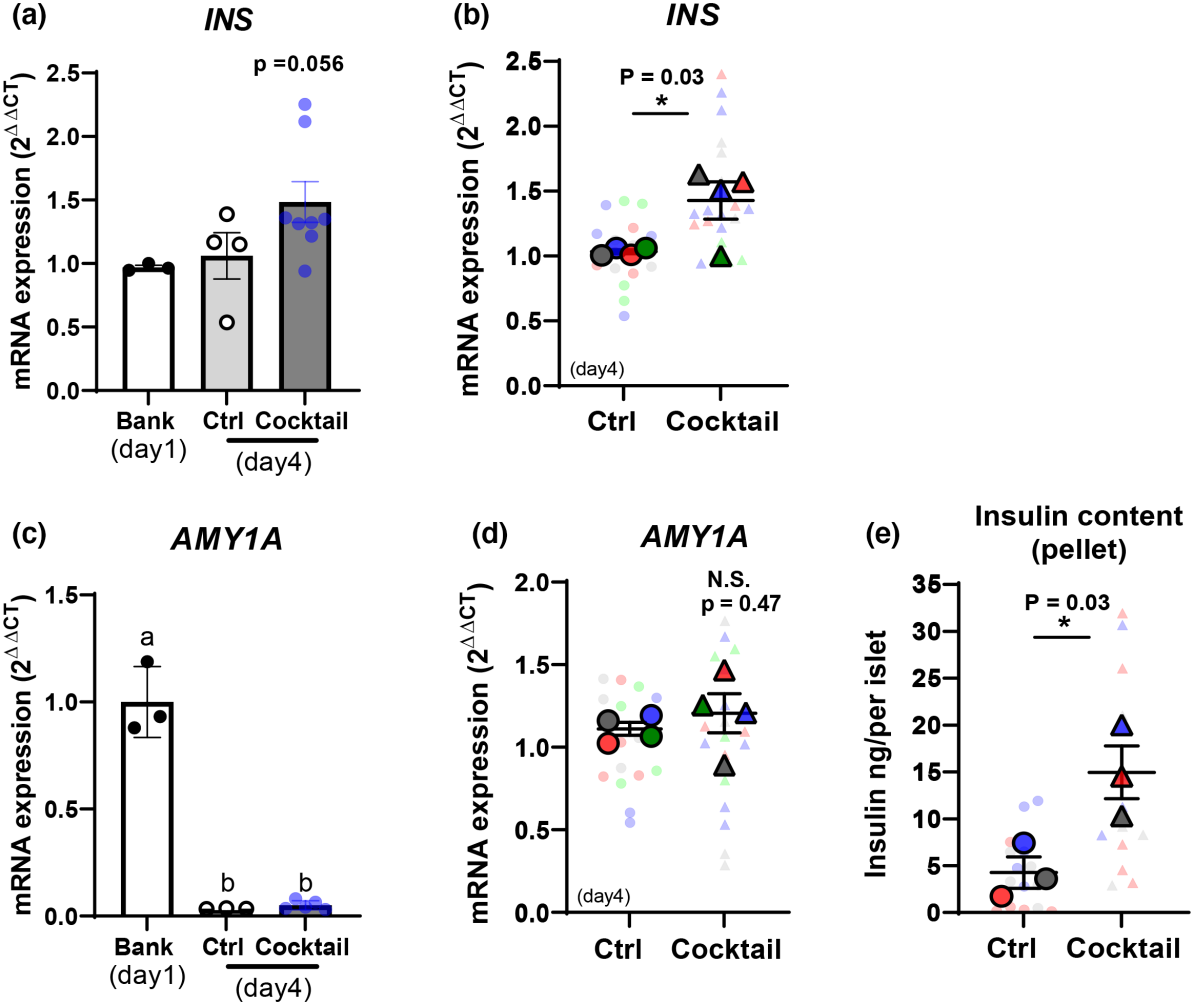
Mesenchymal stromal cell (MSC)‐derived cocktail pretreatment enhances insulin gene expression and content. Human islet extracts were generated immediately post‐isolation (day 1 for tissue bank) and after 72 h in culture (day 4) from untreated islets (control:Ctrl) or from islets treated with the MSC‐derived cocktail. (a, c) Preproinsulin mRNA (*INS*) and amylase mRNA (*AMY1A*) expression were quantified relative to housekeeping genes in islets from donor #5. Cocktail pretreatment enhanced the expression of *INS* mRNA (a) but had no effect on *AMY1A* mRNA (c), which was greatly reduced during 4 days in culture. These data were confirmed in measurements from four islets donors (#5–8) as shown in the Superplots in (b, d). Cocktail pretreatment also increased insulin protein content in the three islet preparations tested (donors #5–7), as shown in panel (e). Data were analysed by Student's unpaired *t*‐tests: *p < 0.05 cocktail pretreated versus untreated control islets. Same colour of two data points represents results from the same human donor as shown in Table 1.

Preproinsulin (PPI) mRNA content of isolated human islets was measured in samples collected on day 1 after isolation (Bank: for reference in our human islet as tissue bank) and on day 4, either in untreated islets maintained in culture (Control) or in islets treated for 72 h with the cocktail of MSC‐derived molecules (Cocktail). Figure 2a shows that PPI mRNA expression relative to the housekeeping genes ACTB was not affected in untreated control islets between day 1 and day 4 (Donor 5, Table 1), but was increased by 72 h treatment with the MSC ‐derived cocktail (Figure 2a *p* < 0.05). Analysis of data from four different islet donors (donors 5–8, Table 1) showed that cocktail treatment induced a significant increase in PPI mRNA expression and insulin protein content (Figure 2b,e). In contrast, expression of amylase mRNA (AMY1A), a marker of exocrine pancreatic cells, was greatly reduced after 72 h in culture (Figure 2c), consistent with the rapid loss of exocrine cells during in vitro culture^29^ and was not affected by treatment with the MSC‐derived cocktail (Figure 2c,d) suggesting the effects of cocktail pre‐culture were specific for the endocrine islet cells.

### 3.3 MSC‐derived cocktail pretreatment induces sustained improvement in human islet graft function in vivo

**FIGURE 3:**
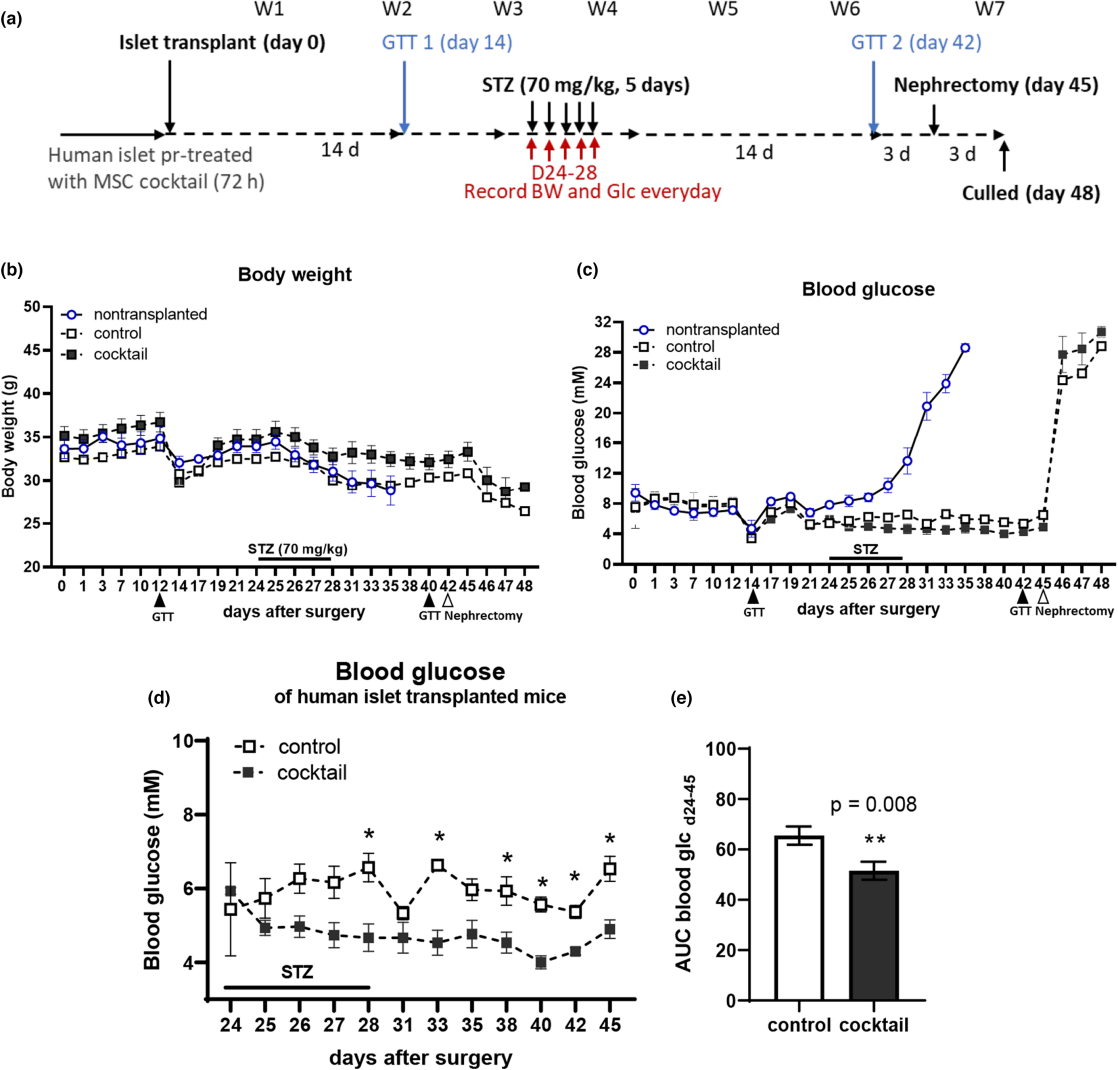
Mesenchymal stromal cell (MSC)‐derived cocktail pretreatment of human islets improves glycaemic control in vivo. The effects of cocktail pretreatment on human islet ability to maintain normoglycaemia in vivo was examined using a mouse model of human islet transplantation. (a) Experimental timeline of the in vivo transplantation study. Glucose tolerance tests (GTTs) were performed 14 days after transplantation and 14 days after STZ administration (arrows; STZ). Nephrectomy was performed on day 45 to remove the human islet graft. (b) Body weight of graft recipients and controls; and (c) basal blood glucose was recorded every other day in the morning. (d) Blood glucose measurements revealed that recipients of cocktail pretreated human islet grafts maintained significantly lower levels of blood glucose from day 24–45 (post‐STZ) when compared to animals receiving untreated human islets (control); (e) this was confirmed by expressing the blood glucose concentrations (day 24–45) as area under curves. Data were analysed by two‐way ANOVA with Bonferroni's post hoc test: *p < 0.05, **p < 0.01 compared to the untreated control islet grafts.

Human islet functional survival in vivo was examined in a mouse model of human islet transplantation (800 islets from donor 5; Table 1), using the experimental schedule shown in Figure 3a. Non‐transplanted control animals showed a sharp decline in body weight (15%) during STZ treatment and 7 days thereafter, whereas both groups of islet graft recipients (non‐treated or pretreated islets) showed more modest weight losses (9% and 6%, respectively) over this period, as shown in Figure 3b. Non‐transplanted control mice also showed marked increases in blood glucose after STZ administration (Figure 3c) compared to both transplanted groups (non‐treated or pretreated islets), demonstrating the successful induction of diabetes by the STZ treatment. These hyperglycaemic animals were sacrificed on day 35, 7 days after STZ administration, in accordance with the ethical guidelines for the use of animals in research. In contrast, animals transplanted with either untreated human islets or islet pretreated with the MSC‐derived cocktail remained normoglycaemic (Figure 3c) for 3 weeks after STZ‐administration, reverting to profound hyperglycaemia within 1 day after graft removal by nephrectomy on day 45. Together these observations demonstrate that the human islet grafts had assumed control of the host blood glucose after destruction of the endogenous mouse beta cells by STZ treatment. Figure 3d,e shows that glycaemia in recipients of cocktail‐treated islet grafts was significantly lower than that of recipients of untreated islets. This is consistent with our detection of human insulin in blood samples taken from the graft recipients at 14 and 28 days post‐transplantation, with recipients of cocktail pretreated human islets having higher levels of circulating insulin compared to recipients of non‐treated islets (untreated islet graft, 0.63 ± 0.4 μU/mL; cocktail‐treated 1.65 ± 0.7 μU/mL; human insulin was not detectable in non‐transplanted controls).

**FIGURE 4:**
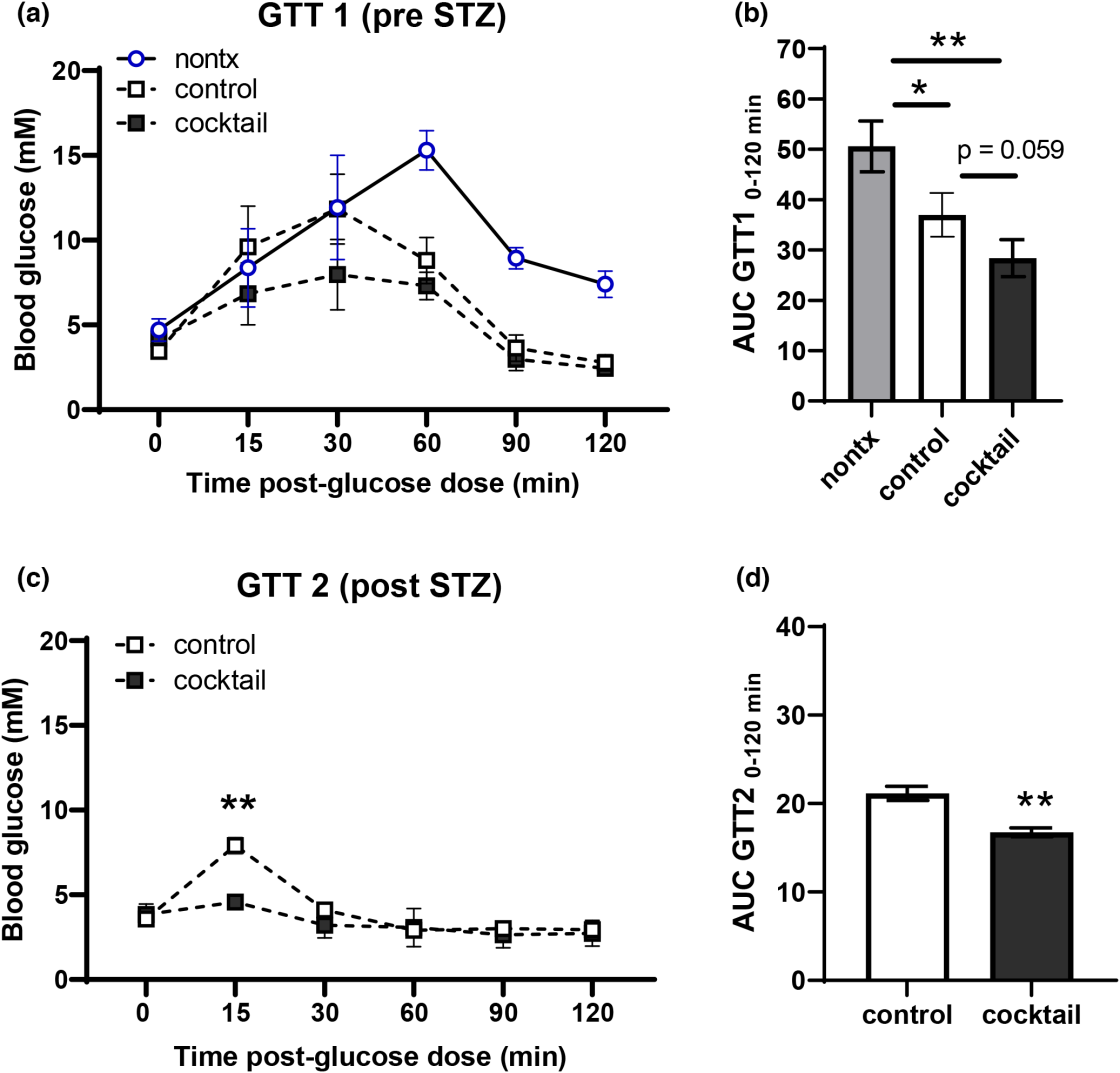
Mesenchymal stromal cell (MSC)‐derived cocktail pretreatment of human islets improves glycaemic control in glucose tolerance tests in vivo. (a–d) Human islet graft function in vivo was assessed by intraperitoneal glucose tolerance tests. (a, b) Human islet grafts significantly reduced peak glucose concentrations in GTTs 14 days after transplantation but before STZ‐administration. Cocktail pretreated human islets were more effective than untreated control islets in maintaining normoglycaemic (*p* = 0.059), as shown in the area under curves analysis (b). (c, d) Human islet grafts were effective at maintaining normoglycaemia in GTTs 42 days after transplantation and 14 days after the induction of endogenous mouse beta cell ablation by STZ administration. Cocktail pretreated human islets were significantly more effective than untreated control islets in maintaining normoglycaemia. Data were analysed by two‐way ANOVA with Bonferroni's post hoc test: *p < 0.05, **p < 0.01 compared to the untreated control islet grafts.

The improved in vivo performance of cocktail‐treated islets was confirmed by the results of the GTTs, as shown in Figure 4. Assessing glucose tolerance 14 days after transplantation, but before the administration of STZ (Figure 4a,b), showed the expected pattern of glucose excursion in non‐transplanted controls. However, in animals receiving human islet grafts the peak blood glucose concentrations were observed much earlier (30 vs. 60 min) with significant improvements in overall glucose tolerance, consistent with the human islets contributing to the responses of the exogenous mouse beta cells to reduce blood glucose. Recipients of the cocktail‐treated human islets showed lower (*p* = 0.059) excursions in blood glucose compared to recipients of non‐treated human islets Figure 4b, suggesting cocktail‐induced improvements in in vivo secretory function. This was confirmed by GTTs performed 42 days after human islet transplantation and 14 days after STZ treatment, at which stage the endogenous mouse beta cells had been destroyed (Figure 3c) and glycaemic control was dependent on the human islet grafts. Thus, Figure 4c,d shows that cocktail treated human islets were significantly more able to suppress glucose excursions when compared to non‐treated human islet grafts. Overall, these observations are consistent with enhanced functional survival of human islets pretreated with the MSC‐derived cocktail and demonstrate that the functional improvements induced in the treated islets were sustained in vivo.

## 4 DISCUSSION

The beneficial effects of co‐culture with MSCs on mouse and human islet function in vitro are well established,^13^, ^14^, ^15^, ^16^, ^30^ and suggest that MSCs may be useful in optimising the outcomes of islet transplantation as a therapy for T1D.^22^, ^31^, ^32^, ^33^, ^34^ We have reported previously that that in vivo co‐engraftment of mouse islets and MSCs in a mouse model of T1D reduced the time to normalisation of glycaemia and reduced the number of islets required to maintain normoglycaemia.^15^, ^16^, ^17^ However, it is technically easy to co‐localise MSCs and islets in mouse models where the site of engraftment is usually under the kidney capsule^13^ but clinical islet transplantation is almost exclusively via the hepatic portal vein, which does not facilitate co‐engraftment of islets and MSCs.^35^, ^36^ Thus, after intraportal delivery the islets (100–200 μm diameter) lodge in the hepatic microcirculation where they re‐vascularise, while the much smaller MSCs (15–30 μm) pass through the liver and most likely end up in the lung microcirculation.^37^ Attempts to co‐localise islets and MSCs by generating islet:MSC composites have shown limited efficacy in vitro with no significant improvement in the in vivo transplantation outcomes in diabetic mice.^38^ We have therefore focused on investigating the mechanisms through which MSCs improve islet graft function to determine whether the beneficial effects can be replicated in cell‐free systems, thus avoiding the requirement to co‐transplant MSCs and also reducing the potential clinical risks associated with MSC transplantation.^18^, ^19^

Our current studies confirm that a defined cocktail of MSC‐secreted GPCR ligands which has previously shown to improve mouse islet functional survival in vitro and in vivo^23^ has similar effects on human islets. The rationale for the inclusion of the individual components of the cocktail has been described in detail elsewhere.^21^, ^23^ In brief, human and mouse beta cells express high levels of the GPCRs for the three components of the cocktail (ANXA1, CXCL12 and C3a); MSCs secrete substantial amounts of all three components^23^; and the individual components enhance insulin secretion or protect from cytokine‐induced apoptosis, or both, in in vitro studies using mouse or human islets.^21^, ^22^, ^23^ In the current study, preincubation of human islets with the cocktail of recombinant human molecules improved glucose‐induced insulin secretion and protected the human islet cells against apoptotic responses to a mixture of inflammatory cytokines. Isolated human islets are inherently much more variable than mouse islets isolated from healthy, young, genetically homogenous mice, as might be expected since factors such as donor age, body mass index, health status and cold ischaemia time are known to affect isolated human islet function.^31^, ^39^ Nonetheless, our use of human islets from nine human donors clearly demonstrated beneficial effects of the MSC‐derived cocktail treatment on human islet function in vitro. We have reported that MSCs are most effective in supporting the function of β‐cells which are compromised by external stressors,^39^ which may explain why some of our human islet preparations were more responsive to the cocktail pretreatment (e.g. donors 5, 8) than others (e.g. donors 2, 3). Future investigations with a larger pool of donors may identify which islet preparations would benefit most from cocktail pretreatment before clinical use as graft material.

The inherent variability in isolated human islet preparations also complicates the assessment of human islet function in animal models in vivo. Most experimental studies with mouse islet grafts use the minimal mass transplantation model in which limited numbers of mouse islets are transplanted into STZ‐induced diabetic mouse recipients to ensure that only ~30%–50% of graft recipients regain normoglycaemia, which enables the assessment of treatment‐dependent improvements in numbers of cured animals, as well as reductions in the time‐to‐cure.^15^, ^16^ The minimal mass model is confounded by the functional variability between different human islet preparations, so we have adapted a “pre‐diabetes” islet transplantation protocol^40^ in which human islets are transplanted into normoglycaemic, immunocompromised mice and allowed to revascularise and engraft before diabetes was induced by multiple low‐dose administrations of STZ. We had previously optimised this model in unpublished preliminary studies to enable the assessment of MSC‐derived cocktail pretreatment on human islet graft function in vivo. The results of this in vivo study in a mouse model of T1D are promising.

Both treated and non‐treated human islet grafts‐maintained host blood glucose levels below normal basal glycaemia in mice, consistent with the lower glucose concentration threshold for human beta cells to initiate insulin secretion.^41^ The differences in beta cell glucose set points between human and mouse beta cells may also explain the earlier peak glucose excursions observed in GTTs in the recipients of human islet grafts before the administration of STZ to ablate the endogenous mouse beta cells. Thus, in non‐transplanted control mice the insulin secretory response to glucose load will be initiated when blood glucose reaches the set point for mouse beta cells (8–10 mM). In the human islet graft recipients, the human beta cells will initiate their secretory responses at lower concentrations of blood glucose (~5 mM), before the endogenous mouse beta cells, causing the earlier return to normoglycaemia seen in our experiments.

The in vivo studies using human islets support a beneficial role for pretreatment of the graft material with the cocktail of MSC‐secreted GPCR ligands. Grafts using cocktail pretreated human islets‐maintained blood glucose at significantly lower levels than non‐treated human islet grafts throughout the post‐transplantation period, consistent with prolonged effects of the pretreatment on improving graft functional survival. Importantly, cocktail pretreated human islet grafts were more effective in reducing blood glucose excursions after intraperitoneal glucose loads, consistent with their elevated insulin mRNA and protein content, and with the enhanced insulin secretory responses of the cocktail pretreated grafts. This is in accordance with similar observations in studies delivering exendin‐4, a GLP‐1 receptor agonist which enhances glucose‐induced insulin secretion, to mice recipients of human islet grafts,^42^ and with our measurements of elevated circulating human insulin in mouse recipients of cocktail pretreated human islet grafts.

In summary, we have demonstrated that pretreating isolated human islets with a defined cocktail of human recombinant MSC‐secreted GPCR ligands prior to transplantation improves their functional survival in vitro and in vivo. We suggest that incorporating this simple treatment into clinical protocols could improve the outcomes of human islet transplantation as a therapy for T1D.

## FUNDING INFORMATION

This work was funded by the Medical Research Council (project grant MR/W002876/1; MRC CARP MR/W030004/1 to SH; MRC DTP studentship to LDG), a David Matthews Research Fellowship from the Diabetes Research and Wellness Foundation to CR. and King’s Health Partners Research & Development Fund.

## CONFLICT OF INTEREST STATEMENT

The authors have no conflicts of interest to declare.

## Data Availability

The data that support the findings of this study are available from the corresponding author upon reasonable request.
